# Advances in Quantitative Earth Remote Sensing: Past, Present and Future

**DOI:** 10.3390/s19245399

**Published:** 2019-12-07

**Authors:** Ghassem R. Asrar

**Affiliations:** Universities Space Research Association, 7178 Columbia Gateway Drive, Columbia, MD 21046, USA; gasrar@usra.edu

**Keywords:** earth observations, multispectral remote sensing, satellites, sensors

## Abstract

A combination of multispectral visible, infra-red and microwave sensors on the constellation of international Earth-observing satellites are providing unprecedented observations for all Earth domains over multiple decades (i.e., atmosphere, land, oceans and polar regions). This Special Issue of ***Sensors*** is dedicated to papers that describe such advances in the field of Earth remote sensing and their applications to advance understanding of Earth’s planetary system and applying the resulting knowledge and information to meet the societal needs during recent decades. The papers accepted and published in this issue convey the exciting scientific and technical challenges and opportunities for remote sensing of all domains of Earth system, including terrestrial, aquatic and coastal ecosystems; bathymetry of coasts and islands; oceans and lakes; measurement of soil moisture and land surface temperature that affects both water resources and food production; and advances in use of sun-induced fluorescence (SIF) in measuring and monitoring the contribution of terrestrial vegetation in the cycling of carbon in Earth’s system. Measurements of SIF, for example, has had a profound impact on the field of terrestrial ecosystems research and modelling. The Earth Polychromatic Imaging Camera (EPIC) instrument on the Deep Space Climate Observatory (DSCVR) satellite located at the Sun–Earth Lagrange Point One, about 1.5 million miles away from Earth, is providing unique observations of the Earth’s full sun-lit disk from pole-to-pole and minute-by-minute, which overcomes a major limitation in temporal coverage of Earth by other polar-orbiting Earth-observing satellites. Active and passive microwave remote sensing instruments allow all-weather measurements and monitoring of clouds, weather phenomena, land-surface temperature and soil moisture by overcoming the presence of clouds that affect measurements by visible and infrared sensors. The use of powerful in-space lasers is allowing scientists and engineers to measure and monitor rapidly changing ice sheets in polar regions and mountain glaciers. These sensors and their measurements that are deployed on major space-based observatories and small- and micro-satellites, and the scientific knowledge they provide, are enhancing our understanding of planet Earth and development of Earth system models that are used increasingly to project future conditions due to Earth’s rapidly changing environmental conditions. Such knowledge and information are benefiting people, businesses and governments worldwide.

## 1. Introduction

The field of Earth remote sensing has been revolutionized during the past several decades due to advances in sensors, satellites and information technologies. This Special Issue of ***Sensors*** is devoted to innovative papers based on multispectral remote sensing measurements of the Earth system enabled by a variety of optical, microwave and laser sensors and a combination of large-, small- and micro-satellites. These measurements are advancing our understanding of the fundamental processes that control Earth’s planetary system, and its components (atmosphere, land, oceans, and polar regions) and their interactions, which in turn control the variability and change of these components and the entire Earth system. The scientific knowledge and resulting data are essential for developing and evaluating the performance of the Earth system models, the only tool available for the prediction of Earth’s weather and climate conditions, water and energy cycles, which have profound socioeconomic importance. For this special issue, we invited papers based on the use of observations from long-term records (e.g., Landsat and Earth Observing System, EOS), multiple satellites (EOS A- and B-Trains, and combined optical and microwave measurements, such as European Copernicus), and satellites in polar, geostationary and other orbits. Studies focused on the innovative use of remotely sensed observations in deriving useful information were also encouraged (e.g., sun-induced fluorescence, vegetation chemistry and composition, species habitat, landscape-level ecosystems genotype and phenotype). We welcomed papers focused on the previous and next generation of Earth-observing sensors and satellites, and/or based on a combination of sensors and satellites (e.g., microsensor/satellite constellations), together with the use of advanced information technologies and systems (e.g., neural networks, artificial intelligence and deep learning). A summary of papers accepted and published thus far is presented in the next section.

## 2. Contributions

We have accepted and published five innovative papers after peer review in this special issue of ***Sensors***. The first paper by Gao et al. [1] describes how Earth Polychromatic Imaging Camera (EPIC) observations can be used for studying large lakes and oceans based on their exchange of solar radiation with atmosphere. EPIC contains ten spectral channels in the 317–780 nm solar spectral range. EPIC data are used in a variety of scientific investigations, including the study of the global ozone levels, atmosphere aerosol index and aerosol optical depth, UV reflectivity of clouds over land and ocean, cloud height over land and ocean, and vegetation indices. In this paper, EPIC observations from narrow channels centered near 443, 551, and 680 nm were used to measure ocean color, an indicator of biological and biogeochemical processes that control the web of life in oceans, for different geographical regions, globally. To remove the effects of intervening atmosphere between the sensor and Earth surface, the authors used a modified version of a multichannel atmospheric correction algorithm for NASA Moderate Resolution Imaging SpectroRadiometer (MODIS) onboard the Terra and Aqua satellites that are also used for monitoring of ocean color. The authors report three case studies on water leaving reflectance retrievals using EPIC observation for a large turbid river, inland lakes, and oceans. They conclude that a future ocean color instrument onboard a satellite at the L1 point, which provides continuous view of the full sunlit disk of the Earth, will complement and extend ocean color observations from the polar orbiting and geostationary Earth-observing satellite, by enhancing current measurements from these sensors/satellites in both spatial and temporal domains.

The second paper, by Yunus et al. [2], presents an improved bathymetric mapping of coasts and lakes using multidecadal Landsat and newly established European Sentinel satellite observations. Data from both satellites are available freely through a variety of portals on the world wide web and the Google Earth Engine, which also offers processing of large datasets, such as the entire Landsat records. The bathymetry of nearshore areas and lakes are affected by the change in sediment transport and dispersal pathways. There is a need for accurate bathymetric models for use in scientific research and management of these areas. Recent advances in space-based observations has revolutionized the availability of bathymetric data, offering new means for mapping of the coasts and lakes, and even remote islands with limited access. This paper is focused on assessing the suitability of Sentinel-2 and Landsat-8 measurements for the bathymetric mapping of coastal and lake areas. The authors used an empirical bathymetric model based on a random forest (RF) model, and the available high-resolution LiDAR bathymetric data for Mobile Bay, Tampa Bay, and Lake Huron for this study. They obtained all reference datasets from the National Oceanic and Atmospheric Administration (NOAA) National Geophysical Data Center (NGDC). Their results illustrate that the satellite-based bathymetry is suitable for obtaining depths of up to 10 m for coastal regions and up to 30 m for the lakes they studied. The root-mean-square error (RMSE) for derived data varied between 1.99 m and 4.74 m for the three cases studied. When they used the RF model, the RMSE for the bathymetric model improved to 1.13 m and 1.95 m. Their comparative analysis suggests that Sentinel-2 has a slight edge over Landsat-8 observations when they used the empirical approach. However, they obtained a better bathymetric model when they used the RF model and Landsat-8 observations as compared with Sentinel-2. This study demonstrates that freely available Landsat 8 and Sentinel-2 observations are suitable for obtaining accurate and up-to-date bathymetric information for relatively large areas in a short period of time.

The third paper, by Tan et al. [3], is focused on the use of all-weather passive microwave remote sensing together with a deep learning convolutional neural network to derive land-surface temperature (LST), which is essential for use in the study and management of water resources and food production. The authors used a convolutional neural network (CNN) algorithm to retrieve LST from brightness temperature measurements by the Advanced Microwave Scanning Radiometer 2 (AMSR2) over China. They used LST from the Moderate Resolution Imaging Spectroradiometer (MODIS) as a reference dataset to overcome any issues related to the comparability of in situ observations in comparison with AMSR2-based LSTs. They randomly divided the AMSR2 brightness temperature (TB) and MODIS LST into training and test datasets, and a CNN was constructed to simulate passive microwave brightness and to invert the surface temperature. When they used the twelve combination of vertical and horizonal (V and H) polarization brightness temperature measurements (7.3, 10.65, 18.7, 23.8, 36.5, 89 GHz), they obtained the most stable and accurate CNN retrieval model. Measurements from V polarizations performed better than H polarizations; however, because CNNs rely heavily on large amounts of data, the combination of both V and H polarizations performed better than a single polarization. The retrievals for different regions revealed that the CNN accuracy was highest over large bare land surface areas. A comparison of the retrieved LSTs with in situ measurements from meteorological stations resulted in R^2^ = 0.987, RMSE = 2.69 K, and an average relative error of 2.57 K.

The fourth paper, by Du et al. [4], is focused on development, test and evaluation of a sensor for in situ measurements of sun-induced fluorescence (SIF), which is used widely in the study of terrestrial ecosystems gross primary production and their contribution to the cycling of carbon in the Earth system. SIF is considered as a proxy for photosynthesis in terrestrial vegetation. In situ and long-term observations of SIF are required for gaining greater insight for seasonal dynamics of photosynthetic activity of ecosystems, including their gross primary production (GPP). The authors describe the design and operation of a tower-based and automated SIF measurement (SIFSpec) system. This system was developed to obtain synchronous SIF observations and flux measurements across different terrestrial ecosystems, and to use these observations for evaluating the satellite-based SIF products (e.g., from European, Japanese and US satellites). The paper provides details about the system components, instrument installation, calibration, data collection, and processing. Correcting for the effects of atmospheric conditions on SIF measurements is also included in their data processing, which is very important but usually ignored for tower-based SIF measurements. They report continuous measurements of SIF for maize during two growing seasons at Daman (DM) flux site (Gansu Province, China) to illustrate the robust performance of their system for capturing the diurnal variations in photosynthetically active radiation (PAR) and seasonal variations in GPP. They obtained very good correlation (0.81) between SIF and seasonal variation of GPP based on measurements from the oxygen-2 (O_2_–A) spectral band. They concluded that automated and continuous SIF measurements with concurrent eddy covariance (EC) flux measurements could provide a reliable approach for understanding the photosynthetic activity of the terrestrial ecosystems, and for better linking of ground-based and airborne and space-borne remotely sensed SIF measurements.

The fifth paper, by Meng et al. [5], is focused on using passive microwave remote sensing to obtain near-surface soil moisture information, which is also essential for the study of water resources and food production. The authors used a spatial fusion downscaling model (SFDM) with Moderate Resolution Imaging Spectroradiometer (MODIS) data to overcome the coarse spatial resolution deficiency associated with passive microwave soil moisture products. They also developed long-term and multi-sensor soil moisture datasets using a time series reconstruction and difference decomposition (TSRDD) method for eliminating the inconsistencies in soil depth and time of measurements among different microwave soil moisture products from the Advanced Microwave Scanning Radiometer on the Earth Observing System (AMSR-E), its successor AMSR2 and the Soil Moisture Ocean Salinity (SMOS). Overall, the downscaled soil moisture (SM) products were consistent with the in situ measurements (R > 0.78) and exhibited a low root-mean-square error (RMSE < 0.10 m^3^/m^3^). The downscaled SM data at a 1-km spatial resolution were used to analyze the spatiotemporal patterns and monitor abnormal conditions in the soil water content across North East China (NEC) between 2002 and 2018. The results showed that droughts frequently appeared in western part of Northeast China and southwest part of the Greater Khingan Range, while drought centers located in the central part of Northeast China. Waterlogging commonly appeared in low lying areas, such as the Songnen Plain. They observed that seasonal precipitation and temperature exhibit distinct interdecadal characteristics that were closely related to the occurrence of extreme climatic conditions and events. Abnormal SM levels were often accompanied by large meteorological and natural disasters (e.g., the droughts of 2008, 2015, and 2018 and the flooding events of 2003 and 2013). The spatial distribution of drought in this region during the growing season indicates that the drought-affected area was larger in the west than in the east, and the semiarid boundary extends eastward and southward.

We have extended the deadline for submission of additional innovative papers to this special issue of ***Sensors*** and will accommodate them until Spring 2020.
